# Differential role of NO-sensitive guanylyl cyclase isoforms NO-GC1 and NO-GC2 in auditory function in adult mice

**DOI:** 10.1186/2050-6511-16-S1-A69

**Published:** 2015-09-02

**Authors:** Dorit Möhrle, Nicole Eichert, Steffen Wolter, Evanthia Mergia, Doris Koesling, Andreas Friebe, Marlies Knipper, Lukas Rüttiger

**Affiliations:** 1 Department of Otolaryngology, Eberhard-Karls-Universität, Tübingen, Germany; 2 Department of Pharmacology and Toxicology, Ruhr-Universität, Bochum, Germany; 3 Department of Physiology, Julius-Maximilians-Universität, Würzburg, Germany

## Background

In the inner ear, elevated cyclic guanosine 3’-5’-monophospate (cGMP) levels were shown to have a sheltering role for cochlear hair cells and hearing function [1]. However, the individual roles of the two nitric oxide-sensitive guanylyl cyclase isoforms (NO-GC1 and NO-GC2) as cGMP generators in this protective effect are still unclear.

The aim of this study was to investigate how the deletion of either one of the α-subunits of NO-GC (NO-GC1 KO or NO-GC2 KO) [2] or deletion of the β_1_-subunit of NO-GC (NO-GC KO), leading to global lack of NO-GC expression [3], affects hearing function, vulnerability to noise exposure and recovery from acoustic trauma in mice.

## Materials and methods

Hearing thresholds and supra-threshold auditory processing at sensation level of NO-GC knockout and wildtype mice were analyzed by measuring the auditory brainstem responses (ABRs). Outer hair cell function was assessed by the distortion product of the otoacoustic emissions (DPOAEs). ABRs and DPOAEs were recorded before and after exposure to intense noise (8-16 kHz, 120 dB SPL, 1 h). Immunohistochemistry was performed on cochlear sections.

## Results

NO-GC1 KO, NO-GC2 KO, and NO-GC KO mice strains showed similar hearing thresholds, outer hair cell function, and amplitudes of ABRs on the level of the auditory nerve fibers, but showed differences in vulnerability to acoustic noise exposure.

## Conclusion

Comparison of the NO-GC knockout with the wildtype mice suggests non-redundant roles of the two NO-GC isoforms in auditory function. The results will be discussed regarding NO-GC as a proposed cGMP generator in functionally distinct parts of the auditory pathway and considering NO/cGMP-signaling as an otoprotective cascade after noise-induced damage of the ear.
